# Gunshot Wound of Kidney

**Published:** 1897-10

**Authors:** W. H. Hudson

**Affiliations:** LaFayette, Ala.


### GUNSHOT WOUND OF KIDNEY. OPERATION. RECOVERY.

By W. H. HUDSON, M.D.,

LaFayette, Ala.

In August, 1896, A. P., aged thirty-two years, a vigorous young white man, was shot with a thirty-two caliber pistol ball.

The ball entered over the left kidney, passed forward and slightly downward, and was lost somewhere in the anterior abdominal wall. Immediately after receiving the wound the man sat down for a moment; but under a strong impulse got up and struggled with his antagonist across the street. A few moments afterward he was carried to his home, one mile and a half way.

Upon reaching home he was in a state of intense collapse, but with the use of stimulants, including two or three hypodermics of morphine, he was revived.

I saw the patient eight hours after he had received the wound. His mind was clear, he had a look of intense agony; his temperature was slightly subnormal, with pulse varying from 140 to 150 beats per minute. The abdominal muscles were contracted; the contraction being so great over the left side of the abdomen as to produce almost wooden hardness. There was also considerable pain on pressure. The legs—especially the left—were flexed toward the abdomen, and could not be straightened without greatly increasing his discomfort. He had passed urine that was highly colored with blood.

From all the symptoms it was evident that the left kidney had been wounded, and that there was more or less intraperitoneal hemorrhage, and it was exceedingly probable that the intestines had been wounded in one or more places.

I placed all the facts of such cases before the wounded man and his family, and advised an immediate operation.

At eight o'clock the next morning—thirteen hours after the wound was received—I operated (Drs. Grady, Heflin, Love and Rea assisting).

The skin of the abdomen had been prepared immediately upon my arrival, and everything to be used during the operation, including the water, had been sterilized by heat.

I made a long incision through the rectus muscle of the left side. The peritoneum at the lower portion of the incision was already adherent to a coil of intestine underneath. Separation of these adhesions revealed a contused spot, evidently made by the bullet, on the concave surface of the descending colon. This contusion appeared not sufficiently deep to require excision and suture, so it was left alone. Further examination showed a large quantity of blood-clot, reasonably estimated at more than a quart, lying in the peritoneal cavity, mostly confined to the left side. A careful examination of the intestines revealed no other wounds. The hemorrhage had evidently ceased to some extent, though there was still bleeding; and the manipulation of the intestines set up again a profuse hemorrhage. The clots were rapidly washed out with hot salt solution, and a hasty effort was made to secure and ligate the bleeding point.

At this time the patient's condition was so weak—he being practically pulseless—that I was compelled to resort to pressure with iodoform gauze to stay the bleeding. The pressure appeared quite effectual, and I decided to depend on it instead of making another effort at ligation, so a yard of iodoform gauze was packed solidly against the region from which the bleeding came, and while being held in this position the peritoneal cavity was carefully washed of all blood with the hot salt solution. The gauze was brought out of the lower angle of the incision, which was then rapidly closed with silkworm sutures, passed through peritoneum, muscles, and skin. Before the operation a quantity of salt solution was injected per rectum, and this was repeated during the operation and afterward. Hypodermics of strychnia also were given.

When the patient was put to bed he was in a state of extreme collapse, but in a few hours he was in a fairly favorable condition. He continued to pass bloody urine until the fourth day, when the blood began to disappear and was entirely gone by the seventh or eighth day. His symptoms were entirely satisfactory until the fourth day, when the gauze was removed. After the gauze had been removed the portion of the peritoneal cavity which the gauze had occupied was freely flushed with warm salt solution, and the patient's condition was considered in every way satisfactory. The bowels had not moved, and in the afternoon a moderate quantity of salts was given. A short time after taking this the patient began to vomit. The vomiting continued for about fifteen hours. I saw him at that time, and administered a large turpentine enema, which produced a free evacuation of the bowels. The vomiting ceased, and with the necessary attention to the abdominal wound the patient's recovery was quite rapid.

He was kept in bed for six weeks, and the abdominal wound now—a year afterwards—is strong, showing no indication of hernia; and the patient's health is good in every respect, there being no indication of kidney disease as a result of the wound.

I have not the literature at hand to make a statistical study of the penetrating wounds of the abdomen in which operations have been made; but there are certain well established facts which are to guide the physician and surgeon in their treatment of such cases. When it is fairly probable that the stomach or intestine, or indeed any of the hollow viscera have been penetrated, it is their imperative duty to open the abdomen and repair the damages, or by drainage or the staying of the bleeding to place the patient in such condition as to make it possible for him to recover.

In cases of gunshot wounds it is quite probable that the bullet has taken the course indicated by the wound of entrance, and if the bullet has passed into the abdominal cavity, some portion of the intestinal tract has, with almost absolute certainty, been penetrated.

Wounds of the kidney, without other complications, need not necessarily prove fatal; but of course the difficulty in such a case would be to decide whether there was not more injury than the mere wound of the kidney. If there was extravasation of urine it should be drained from the back, and the drainage carried well up to the kidney. It might, in certain cases, be necessary, to remove a kidney which had been injured to such an extent as to render the repair improbable, or to endanger the patient's life by prolonged suppuration. But we should bear in mind that the reparative power of the kidney is great, and a just chance should be given in each case.

Injuries of the liver and spleen should be managed from the same standpoint, controlling hemorrhage when possible by deep suture, or if necessary by packing with sterile weak iodoform gauze; in wounds of the spleen the bleeding may be so dangerous and uncontrollable as to necessitate the removal of this organ.

In a large majority of the cases in which operations have been done for perforating wounds of the abdominal cavity, the incision has been made in the median line. I see no reason, however, why the incision should not be made at any point which would most conveniently expose the injury.

If there has been excessive hemorrhage, or if it still continues, it should first be stopped, and then the perforations sought for and closed.

The peritoneum should be carefully cleansed, the exudation from every perforation carefully and rapidly removed before the peritoneal cavity has been flushed. Indeed, if there has been only one or two perforations with little or no effusion of the intestinal contents, it may be wisest not to flush the peritoneal cavity at all. At any rate the fluid used for flushing should always be hot sterile salt solution, preferably poured in from a pitcher or large bottle; and if drainage is used it is best done by inserting strips of sterile weak iodoform gauze in different directions.

These cases of perforating abdominal wounds are necessarily in a bad condition, and an operation, if done, should be done early, rapidly and thoroughly, the patient being fortified by a small quantity of morphine given hypodermically, strychnine in larger quantities given the same way, and salt solution injected into the veins and into the rectum, and in certain cases left in the peritoneal cavity.

A word in reference to the symptoms: As in many other diseases involving the contents of the abdominal cavity, the symptoms may be very deceptive; for instance, there may be several perforations of the intestines, and yet the accompanying signs may be very insignificant for many hours after the injury; or, as in a case which I saw several years ago, the bullet may have struck a button and only produce a blue spot on the skin, and yet the man was in extreme collapse with very rapid pulse, wet skin and with every indication of very serious injury. Usually, however, in these cases in which the injury is severe the symptoms are indicative and point with great certainty to the dangerous condition of the patient.

These patients should be carefully examined, and unless we are very certain that none of the hollow viscera have been penetrated, and that there is no great amount of hemorrhage from the solid viscera, or any blood-vessel, an operation should be done and that at the very earliest moment.

